# Drivers of perceived discrimination among older adults in India: an intersectional analysis

**DOI:** 10.1186/s40359-024-01697-7

**Published:** 2024-04-10

**Authors:** Jayantika Chakraborty, Sampurna Kundu

**Affiliations:** 1 Frances L. Hiatt School of Psychology, Clark University, Worcester, Massachusetts United States; 2 Centre of Social Medicine and Community Health, School of Social Sciences, Jawaharlal Nehru University, 110067 Delhi, India

**Keywords:** Caste, Gender, Economic condition, Discrimination, India

## Abstract

Discrimination is harmful action taken against individuals or groups to protect customary relations of power and privilege. Older adults are particularly vulnerable to experiences of discrimination that adversely affect their quality of life. We use data from the Longitudinal Ageing Study of India (LASI; Wave 1; 2017–2018) to examine different contextual forces that shape the experiences of discrimination in older adults in India, specifically gender, caste, and economic condition. We used the theory of intersectionality to hypothesize that economic condition, caste, and gender combine uniquely to engender perceived discrimination in older adults. We first used a concentration index to determine the sample’s pre-existing inequality levels. The concentration curve evidenced a disproportionate concentration of discrimination among people with low income. Next, we used a three-way ANCOVA to examine the effects of caste, gender, and economic condition on individuals’ experiences of discrimination. A significant interaction effect of caste, gender, and economic condition [*F*(1, 30,394) = 8.91 *p* = 0.003] evidenced the compounding effects of inequalities on experiences of discrimination. Finally, we ran a moderation model to test the ameliorating effects of education on experiences of discrimination experienced by marginalized castes. The model was significant (β= -0.192; *p* < 0.001), thereby supporting the proposition that increased education level can lead to an increased sense of belonging and perceptions of equal treatment, which relate negatively to perceived discrimination. Results are discussed considering intersectionality in peoples’ struggles and resilience in India.

## Drivers of discrimination in India: an intersectional analysis

The disparity in global knowledge production is quite disproportionate, with most funded knowledge produced by rich and powerful nations in the global north [1]. The psychological reality of the global south is hardly, if ever, adequately represented in the psychology literature. Yet, the complex reality of the global south offers rich opportunities for investigating different psychological domains that could propel and foster theoretical developments and even make space for pre-existing theories to account for greater variability. India’s diversity, plurality, and multiculturalism present a unique opportunity to test the effects of different cultural forces on experiences of discrimination and marginalization. However, there is a gap in the literature when it comes to understanding the intersection of different factors that shape experiences of discrimination in India. Thus, in the current paper, we used data from the Longitudinal Ageing Survey of India (LASI; Wave 1; 2017–2018) to examine the different contextually relevant forces that shape the experiences of discrimination in older adults in India. More specifically, the three contextual factors we examined are gender, caste, and economic condition. Moreover, we also examined education level as a potential buffer between caste affiliation and perceived discrimination, thereby highlighting the importance of education as a tool to diffuse discrimination by increasing a sense of belonging.

### Experiences of discrimination in India through an intersectional lens

Social groups are hierarchically self-organizing units where members vary in power, influence, skill, or dominance [2]. India is a demographically diverse country with the seventh-largest geographical area and the second-largest population [3]. This demographical diversity lends itself to group-based hierarchical social organizing. Such a system of organization in Indian society leads to steep power asymmetries between the differently positioned stakeholders in the hierarchy. Although experiences of discrimination are based on one’s identity vis a vis their hierarchical positioning in a particular group, the existence of multiple groups leads to people identifying themselves with multiple groups simultaneously. In such circumstances, one can concomitantly be at a disadvantageous position in the hierarchy of one group (e.g., lower caste; Shudra; see below) and at an advantageous position in the hierarchy of another (e.g., gender; men). Such intersections of differential identities leading to qualitatively different experiences of discrimination and privilege are captured by the theory of intersectionality [4].

The theory of intersectionality withholds the idea that multiple identities interact with one another instead of existing separately, in which the most oppressed are always the most salient [5]. The theory was born out of the need to understand the structural barriers Black women face in the United States [4, 6]. Despite feminist and antiracist theories addressing oppression and marginalization, they were not adequately capturing the unique experiences of Black women or the “double discrimination” experienced by them for their gender and their race. Crenshaw (1989, 1991) specifically examined the oppression of Black women in court trials, in records of rape [4] and in domestic abuse [6]. The theory of intersectionality has been used to understand marginalized women’s experiences in different contexts- in educational leadership roles in England, South Africa, and the United States [7], in addressing intersectional issues like climate change [8], in deconstructing the politics of migration and transnational mobility [9], to name a few. Furthermore, the intersectional approach addresses identity multiplicities and heterogeneities and pushes the envelope beyond single-issue politics, as proposed by Black feminist scholarship [10].

Given the plurality of India, it is necessary to employ an intersectional lens to unpack, understand, and contextualize the discrimination endured by the marginalized and disenfranchised in the subcontinent. However, Menon (2015) cautions against the thoughtless transplantation of intersectionality in a postcolonial context like India [11]. An examination of the Indian context using an intersectional approach would entail the investigation of caste as a co-constituting reality in the matrix of marginalization, along with gender and economic-condition-baseddiscrimination. Although caste shares many parallels with racial apartheid [12], it is primarily based on a system of descent and is one of the most pervasive parameters dividing Indian society [13]. Thus, the current paper will examine the relationship between gender, caste, and economic condition and tease out how each of these distinctly and conjointly affects experiences of discrimination among older adults in India.

### Experience and repercussions of discrimination among older adults

Discrimination is harmful action taken against individuals or groups to protect customary relations of power and privilege [14]. Discrimination need not always be intentional by the privileged against the minoritized since stratification systems often produce routine actions with no direct intention to harm but which can still be discriminatory to entire communities [15]. Perceived discrimination occurs when an individual infers that they are a target of discriminatory actions, either intentional or unintentional [16]. This perceptual process is influenced by social status, autobiographical experiences, emotions, and mental health conditions [17–20].

Discrimination, especially when perceived, takes an emotionally taxing toll on the mind of its victims, with experiences of discrimination being related to the higher frequency of psychiatric disorders and distress [21], poorer health [22], and compromised cognition [23]. Discrimination is associated with poor mental health, including mood disorders, depression [24, 25], psychological distress [26], anxiety [21]; and general stress [15]. Finally, discrimination has an adverse impact on chronic illnesses like kidney failure [27] and leads to an increased mortality risk [22]. Thus, the accrual of life stressors and discrimination places older adults on the vulnerable verge of diminished quality of life.

Although the effects of perceived discrimination on older adults’ quality of life are robustly established in the literature, there are some crucial gaps. First, the literature on the psychological ramifications of perceived discrimination in older adults is limited (see Barnes et al., 2008 and Sutin et al., 2015, for some documentation) [22, 28]. Second, there is a limited representation of populations from the global south in documenting the drivers of perceived discrimination. Finally, there is a void in empirical work to understand discrimination from an intersectional lens in India.

### Gender-based discrimination in India

The persistence of inequality between men and women is more pronounced in Asia than in any other part of the world [29]. Drèze & Sen (2002) recognized this inequality and resulting discrimination between men and women as one of the most crucial and cardinal disparities in most Indian societies [30]. Gender discrimination in India is a product of cultural features that exacerbate male favoritism [31] and cherishes the idea of male domination in most spheres of life, including the workplace [32]. Such socially cherished ideas of male superiority lead to spillover effects of gender-based discrimination in the organizational workforce, social and political contexts [33]. Gender discrimination in India is manifested in different sectors. First, the education sector witnesses gender discrimination as evidenced by parents’ pro-male bias in educational investment [34]. Women face poor economic incentives to pursue education than men since they are believed to reap lower labor market returns to education than males. A few sociocultural mechanisms that prevent access to education for women are the dimensions of caste, religion, economy, ethnic origin, or simply the color of their skin [35]. Gender discrimination is also widely prevalent in the labor force, with widening wage gaps and restricted access to male-dominated spaces. According to the World Economic Forum, Indian women are paid 62% of what their male counterparts earn for the same position and equal work [36]. Sankaran & Madhav (2011) identified how unequal gender relations affect women in the workplace: minimal negotiation power and poor representation, lack of control over work-life balance, minimal family support, limited access to institutional support, and inequality in financial literacy and educational resources [37]. Overall, substantial gender-based discrimination in India is evidenced overtly and covertly through institutional practices and cultural endorsement of unequal gender dynamics.

It is worth noting that participation of women in the Indian workforce demonstrates a *U* effect where workforce participation of illiterate women from rural areas is high; workforce participation for women with low and intermediate education is low; and there is an upward trend for women with graduate or postgraduate degrees [38]. Therefore, education is not only a tool for social mobility but also a catalyst through which women can demonstrate their agentive participation in the workforce.

### Caste-based discrimination in India

Caste-based discrimination in India is a product of deeply ingrained belief in Aryan racial supremacy. In the caste system, upper caste members claim a superior lineage by tracing their “genes” to Aryans, implying a “natural superiority” over Shudras (the socio-legal term for Shudras is Scheduled Castes) [39]. Caste classification is order of increasing social disadvantage is General Caste, Other Backward Caste (OBC), Schedule Caste (SC), and Schedule Tribe (ST). SC and ST castes primarily comprise Dalits and Adivasi people respectively [40, 41]. Dalits (literal meaning ‘broken’) are historically accorded jobs like manual scavenging, floor-sweeping, and street-cleaning, thereby being referred to as *Untouchables*. Adivasis are the indigenous people of India.

Textually, physical differences characterize the difference between upper castes and Shudras; the former are described to be of lighter skin color, and the latter racially inferior owing to their dark skin. The *varna-jati* system in India perpetuates such a social hierarchical practice of discrimination. Present-day manifestations of such discrimination are reflected in restricted commensality, endogamy, rules of dining, and practices of untouchability which, in fact, are banned by the constitution of India (equality before the law, Article 14; social equality and equal access to public areas, Article 15). Yet, caste-based discrimination is widely witnessed and is recognized as a feature of the Indian labor market and business economy [42]. In the current paper, we will refer to the OBC, SC, and ST as the marginalized castes.

### Economic condition-based discrimination in India

Socioeconomic status (SES) is defined as an assessment of an individual’s overall economic and social standing [43, 44]. Generally, SES is conceptualized as a latent construct, and its measurement involves a composite index comprising education, income, and occupation, or variations thereof, as key indicators (Baker, 2014). The utilization of proxy indicator variables to assess SES is widespread in both social and behavioural research contexts, from individual indicators (such as parental income on an annual or monthly basis) to comprehensive scales (such as the Duncan Socioeconomic Index; Cabrera et al., 2018) [45]. In the current study, we have utilized one’s monthly per capita expenditure as a proxy forSES. Thus, SES in the current study is a measure of economic affordances, which serves as an indicator of socioeconomic inequality. In the interest of keeping our language consistent, we will use the term ‘economic condition’ in the rest of the paper.

Discrimination based on economic condition in India cannot be discussed in isolation since the parameters that characterize such discrimination are driven by caste and gender. It is, therefore, crucial to understand the “grammar” of caste [46] behind persisting economic disparities in India. India’s capital wealth (land, buildings, finance, etc.) is largely in the hands of the “upper” (General) castes, while the “lowest” (marginalized) castes are primarily relegated to wage laborers in the economy [42]. As we move down the caste hierarchy, per-capita income or access to high-status occupations is limited, as does the return on factors like education and capital assets, thereby contributing to a concentration of poverty in the sphere of the marginalized castes. Dalit leader B.R. Ambedkar referred to this system as one of “graded inequality” [47]. Aggregating the different disparities in occupation, education, and assets into a Caste Development Index, Deshpande (2013) has demonstrated that the degree of caste-based inequality has unimproved and sometimes worsened by faster growth of different Indian states [46]. Therefore, discrimination based on economic inequality in India is engendered by caste-based discrimination, which is further compounded by gender-based discrimination. In a study on perceived discrimination among pregnant women in rural India, Khubchandani et al. (2018) found that in comparison to “upper” caste pregnant women, “lower” caste pregnant women were more likely to experience discrimination, accept discrimination, and keep to oneself about discrimination. Such adverse experiences of discrimination based on gender and caste are likely to bleed into experiences of economic condition-based discrimination since “lower” or marginalized castes are more likely to be working in low-wage, poorly secure jobs, as reported above [48].

### Discrimination and social exclusion

Experiences of discrimination lead to social exclusion. Social exclusion can be of two kinds- one, where individuals are kept out (or left out), and the other, where circumstances of inclusion are on deeply unfavorable terms [29]. Either type can generate adverse effects. Pervasive perceived discrimination affects psychological well-being (self-esteem, depression, anxiety, psychological distress, and life satisfaction) across the lifespan [49]. Perceived discrimination also produces heightened stress responses and is related to participation in healthy and non-participation in unhealthy behaviors [50]. A review of caste exclusion and health discrimination in Southeast Asia by Thapa et al. (2021) revealed that caste-based inequity impacts all aspects of an individual’s well-being, including violence and risk-taking behaviors [51]. Moreover, caste also impacts individuals’ opportunities to access education, employment, and health care. Marginalized castes and women belonging to marginalized castes experience the effects of this inequality more prominently due to their disadvantageous economic condition, caste status, and gender, which play a combined role in their increased vulnerability to health risks.

### Perception of discrimination and education

Discrimination, or the perception of being treated unfairly due to certain personal attributes, is unequally experienced by individuals within various population subgroups. Perceived discrimination may result from belonging to a combination of social identities, such as gender, caste, and economic condition, rather than any single identity. Additionally, the cultural norms within a social context may impact the likelihood of certain social identities becoming targets for discrimination, especially among population subgroups adopting new roles and accessing resources like education and employment in an emerging knowledge economy [52]. Perceiving discrimination in both overt (e.g., hostility, neglect, physical and emotional abuse) and covert (e.g., benevolent sexism, microaggressions) forms is essential to form an informed understanding of discrimination and promotes increased support for political rights, activism, and activism intentions (e.g., Stronge et al., 2015; Cronin et al., 2012; Smith & Williamson, 2020; respectively).

Population subgroups do not homogenously experience the perception of discrimination by individuals. Individual-level differences, like people’s sensitivity to injustice, can lead to differential experiences in perceiving discrimination. Higher education attainment can lead to either increases or decreases in individuals’ perception of discrimination. On one hand, education can play a role in increasing people’s awareness of discrimination and injustice (e.g., American Civil Liberties Union, 2023) [53]. Indeed, higher education attainment by small immigrant groups in the Netherlands (i.e., Afghani, Iraqi, Irani, Somali, Polish, and Chinese) led to experiences of more discrimination by these groups than lower-educated immigrants [54]. Similarly, higher education among Polish and Turkish immigrants in Germany was associated with higher levels of perceived discrimination [55].

On the other hand, educational attainment can lead to upward social mobility where educated individuals’ sense of belonging and perception of equality might temper their perception of discrimination. Indeed, education fosters a sense of belonging in students (e.g., Parkes, 2014) [56]. Moreover, the sense of belonging fostered by educational pursuits may be accompanied by social engagement [57]. Feelings of social engagement might positively correlate with experiencing success [58], which might take precedence over perceptions of discriminatory actions at the workplace.

In conclusion, higher education has the potential to enhance awareness of discrimination and injustice, thereby leading to increased experiences of discrimination among minoritized groups. At the same time, higher education can also foster a sense of belonging and social engagement, which may mitigate perceptions of discrimination, especially in environments where individuals experience professional success. Thus, the impact of higher education on perceptions of discrimination is complex and multifaceted, influenced by various factors including individual differences, societal norms, and the context in which the education is pursued.

### The current study

India’s diversity, plurality, and multiculturalism present a unique opportunity to test the effects of different cultural forces on experiences of discrimination and marginalization. However, there needs to be more literature to understand the intersection of different factors that shape experiences of discrimination in India. Thus, in the current paper, we will use data from the Longitudinal Ageing Survey of India (LASI; Wave 1; 2017–2018) to examine the different contextually relevant forces that shape the experiences of discrimination in older adults in India. More specifically, the three contextual factors we will examine are gender, caste, and economic condition. Moreover, we will also examine education level as a potential buffer in experiencing discrimination. Educational attainment can lead to perceptions of equal treatment, thereby serving as a shield of protection from the adverse effects of marginalization [59]. This conceptualization served as the impetus for us to examine education as a buffer between caste and perceived discrimination.

Our research questions, rationale for statistical techniques, and hypotheses are as follows:

1. Is there a disproportionate concentration of perceived discrimination among older adults from disadvantageous or low economic condition backgrounds? We will use a concentration curve and concentration index to answer this research question. The concentration curve and index allow for a direct comparison between groups of different economic conditions and is a robust method for investigating economic disparities in the population [60]. We hypothesize a higher concentration of perceived discrimination by older adults from low economic condition backgrounds relative to those from high economic condition backgrounds.
2. Do gender, caste, and economic condition interact to produce differences in the experiences of discrimination in older adults? To answer this question, we will use an Analysis of Covariance (ANCOVA) model with gender, caste, and economic condition as independent variables and perceived discrimination as the dependent variable. We will control for marital status, religious affiliation, residency status, age, and education. We hypothesize that gender, caste, and economic condition will intersect to produce the highest levels of perceived discrimination for women from marginalized castes and who belong to low economic backgrounds.
3. Does education moderate the relation between caste and discrimination, wherein older adults from marginalized castes who have received higher education experience lower levels of discrimination than those who have not received higher education? We will use a moderation model to answer this research question. We will control for age, gender, residence, religion, economic condition, and marital status. We hypothesize that education will moderate the relation between caste and perceived discrimination, such that older adults who belong to marginalized castes and have received higher education will report lower levels of perceived discrimination.

## Method

### Data source

The present study utilizes the Longitudinal Aging Study in India (LASI) Wave 1, 2017–2018, coordinated by the Harvard T.H. Chan School of Public Health, the International Institute for Population Sciences, Mumbai, and the University of Southern California. The survey has gathered significant data on the physical, social, and cognitive health of people (72,250) in all Indian states and union territories (with the exception of Sikkim) who are 45 years of age and older. A multistage stratified area probability cluster sampling design was utilised for the survey, with a three-stage sampling design for rural areas and a four-stage sample design for urban areas. (LASI, 2020).

The study has restricted our population to 60 and above with a sample of 31,464 individuals, consisting of men (14,931) and women (16,533). Our study did not require any approval by the ethical review committee as the data is publicly available. Total sample size is 31,464, with less than 5% missing data [61].

### Variable description

#### Outcome variable

An individual’s opinion that they have been subjected to unjust treatment by others because of their color, ethnicity, age, gender, socioeconomic condition, sexual orientation, or other qualities is known as perceived discrimination [62, 63]. Because older adults who internalize unfavorable attitudes about themselves are more likely to experience functional and cognitive deterioration, it is important to measure reported daily discrimination among this population [64, 65]. Thus, to measure discrimination experienced every day by older adults, LASI included six statements that include- a) one is treated with less courtesy or respect; b) receives poorer service than others at restaurants or stores; c) one is made to feel like that people think he/she is not smart; d) one is made to feel like people act as if they are afraid of him/her; e) one is threatened or harassed, and f) one received poorer service or treatment than other people from doctors or hospitals. The responses had six categories: ‘almost every day,’ at least once a week,’ a few times a month,’ a few times a year,’ ‘less than once a year,’ or ‘never.’ Never was coded as ‘0’, and the rest was coded as ‘1’ for each of the six statements. A composite index was generated with scores ranging from 0 to 6. The Cronbach’s alpha was 0.87, indicating excellent internal consistency.

#### Independent variables

To observe how belonging to a vulnerable or marginalized caste can lead to discrimination, caste is taken as the independent variable. There are four categories for caste- scheduled caste (SC), scheduled tribe (ST), other backward classes (OBC), and others. Belonging to SC, ST, or OBC is coded as ‘1’ and others as ‘0’. The other socio-demographic variables in the study, which includes age (60–116), gender (men, women), residence (urban, rural), marital status (married, non-married (including widowed/divorced/separated)), and religion (Hindu, non-Hindus). The monthly per capita expenditure (MPCE) quintile measured the economic condition using household consumption data. Sets of 11 and 29 questions on the expenditures on food and non-food items, respectively, were used to canvas the sample households. Food expenditure was collected based on a reference period of seven days, and non-food expenditure was collected based on reference periods of 30 days and 365 days. Food and non-food expenditures have been standardized to the 30-day reference period. The monthly per capita consumption expenditure (MPCE) is computed and used as the summary measure of consumption. MPCE was classified into five quintiles: poorest, poorer, middle, richer, and richest (LASI, 2020).

#### Moderator

Educational level is hypothesized to moderate the effect of caste on perceived discrimination. The level of education has four ordered categories: 0: ‘no education,’ 1: ‘completed primary,’ 2: ‘completed secondary,’ and 3: ‘completed diploma/college.’

### Statistical techniques

The variables considered in the study were first described using summary statistics, that is, mean, standard deviations (continuous variables), frequency distribution, and percentages (categorical variables). Bivariate analysis was carried out to examine the significant association between the moderator, possible cofounders, and the dependent variable: perceived discrimination. Independent t-tests were used for categorical variables with two categories and one-way ANOVA F-test for more than two categories. The effect sizes and p-values are also reported.

Economic inequality in facing discrimination among older adults was quantified by the concentration index (CCI) and the concentration curve (CC), using the household wealth score as the economic indicator and perceived discrimination as the binary outcome variable. The concentration curve is obtained by plotting the cumulative proportion of older adults who experienced discrimination against the cumulative proportion ranked by the economic indicator [66, 67]. The concentration index can be written as follows:

$$ C=\frac{2}{\mu }cov\left({y}_{i,}{R}_{i}\right)$$

where C is the concentration index; $$ {y}_{i}$$ is the outcome variable index; ***R*** is the fractional rank of individual ***I*** in the distribution of economic position; $$ \varvec{\mu }$$ is the mean of the outcome variable of the sample, and $$ \varvec{c}\varvec{o}\varvec{v} $$denotes the covariance. If the curve lies above the line of equality, the concentration index takes a negative value, indicating a disproportionate concentration of inequality among people with low incomes. Conversely, if the curve lies below the line of equality, the concentration index takes a positive value, indicating a disproportional concentration of inequality among the rich. In the absence of economic condition-based - inequality, the concentration index is zero.

To test the second hypothesis, we used a 2 × 2 × 2 ANCOVA. The three fixed factors in this 3-way ANCOVA model are economic groups (A), caste (B), and gender (C). Let Y_ijkt_ denotes the outcome variable at the t^th^ observation at i^th^ level of A, j^th^ level of B, and k^th^ level of C. The 3-way ANOVA model is denoted by the following complete model equation

$$ {Y}_{ijkt}=\mu +{\alpha }_{i}+{\beta }_{j}+{\left(\alpha \beta \right)}_{ij}+{\left(\alpha \gamma \right)}_{ik}+{\left(\beta \gamma \right)}_{jk}+{\left(\alpha \beta \gamma \right)}_{ijk}+{\epsilon }_{ijkt}$$

where ${\epsilon }_{ijkt}$ are independent error terms following a normal distribution with zero means and constant variance; ${\left(\alpha \beta \gamma \right)}_{ijk}$ is the 3-way interaction term.

Following the guidelines provided by Preacher and Hayes (2008), the moderation hypothesis has been tested [68]. Based on 5000 bias-corrected bootstrapped samples, the 95th percentile confidence interval for the mediation analysis was generated using SPSS. The following equation makes up the model’s analysis of the relationship between caste (X) and discrimination (Y), which is moderated by educational attainment (M)

1 $$ Y={i}_{y}+aX+bM+cXM+{\epsilon}_{1}$$

where i_y_, is the intercept; a is the effect of X on Y; b is the effect of M on Y; c is moderation effect of M. This is a common method used in many social science research [69, 70].

## Results

The mean age of the study population is 69 years, ranging from 60 to 116. Around 53% of the sample is composed of women and the rest 47% are men. The majority of the older adults were Hindu (82.22%) and lived in rural areas (70.55%). More than half of the sample was married (61.63%). The older adults belonging to the poor economic quintile were around 22%. About 27.7% of the sample belongto marginalized castess. More than half of the sample (56.52%) had completed a diploma or college, while 22.6% had received no schooling. The discrimination index shows a mean value of 0.44 for the sample, ranging from 0 to 6 (Table 1).

**Table 1:** Descriptive statistics of the study variables (*N* = 31,464)

		*n*(%)	Mean(S.D)	Range
**Covariates**				
Age			69.18(7.53)	60–116
Gender	Male	14,931(47.45)		
	Female	16,533(52.55)		
Residence	Rural	22,196(70.55)		
	Urban	9268(29.45)		
Marital status	Not married	12,073(38.37)		
	Married	19,391(61.63)		
Religion	Non-Hindu	5593(17.78)		
	Hindu	25,871(82.22)		
MPCE	Poorest	6829(21.7)		
	Poorer	6831(21.71)		
	Middle	6590(20.95)		
	Richer	6038(19.19)		
	Richest	5175(16.45)		
**Independent variable**				
Caste	Others	22,735(72.26)		
	SC/ST/OBC	8729(27.74)		
**Mediator**				
Education	No schooling	7118.2408(22.62)		
	Completed primary	5285.04785(16.8)		
	Completed secondary	1277.7305(4.06)		
	Completed diploma/college	17782.981(56.52)		
**Dependent variable**				
Discrimination index			0.44(1.19)	0–6

*Note* SC, ST, and OBC stand for Scheduled Castes, Scheduled Tribes, and Other Backward Castes, respectively; MPCE stands for Monthly per capita expenditure

The bivariate results in Table 2 show the average discrimination scores across the study variables. The discrimination score is significantly higher among the rural residents (0.43; *p* < 0.001; d = 0.06). For those who are not married, the discrimination score is higher (0.432; *p* < 0.01; d = 0.04). Discrimination score is also higher among Hindus (0.44; *p* < 0.001; d = 0.12), the poorest economic group (0.44; *p* < 0.05; d = 0.02), belonging to marginalized castes (SC/ST/OBC) (0.42; *p* < 0.001; d = 0.06) and is significantly associated with these variables. However, no significant association was observed for discrimination with age and gender.

**Table 2:** Bivariate tests of covariates and mediator with discrimination index

		Discrimination score			
		*Mean(S.D)*	*Test*	*p-value*	*Effect size*
Age			*r* = 0.006	> 0.001	
Gender	Male	0.398 (0.009)	t= -0.684	> 0.001	0.01
	Female	0.408 (0.009)			
Residence	Rural	0.427 (0.008)	t = 4.947	< 0.001	0.06
	Urban	0.357 (0.011)			
Marital status	Not married	0.432 (0.011)	t = 3.216	< 0.01	0.04
	Married	0.387 (0.008)			
Religion	Non-Hindu	0.290 (0.011)	t= -11.316	< 0.001	0.12
	Hindu	0.444 (0.008)			
MPCE	Poorest	0.443(1.196)	F = 2.41	< 0.05	0.001
	Poorer	0.386(1.139)			
	Middle	0.393(1.135)			
	Richer	0.400(1.181)			
	Richest	0.396(1.175)			
**Independent variable**					
Caste	Others	0.353 (0.011)	t = 4.922	< 0.001	0.06
	SC/ST/OBC	0.424 (0.008)			
**Moderator**					
Education	No schooling	0.354(1.076)	F = 48.95	< 0.001	0.004
	Completed primary	0.304(1.039)			
	Completed secondary	0.225(0.92)			
	Completed diploma/college	0.475(1.254)			

*Note* SC, ST, OBC stand for Scheduled Castes, Scheduled Tribes, and Other Backward Castes, respectively; MPCE stand from Monthly per capita expenditure

**Fig. 1:**
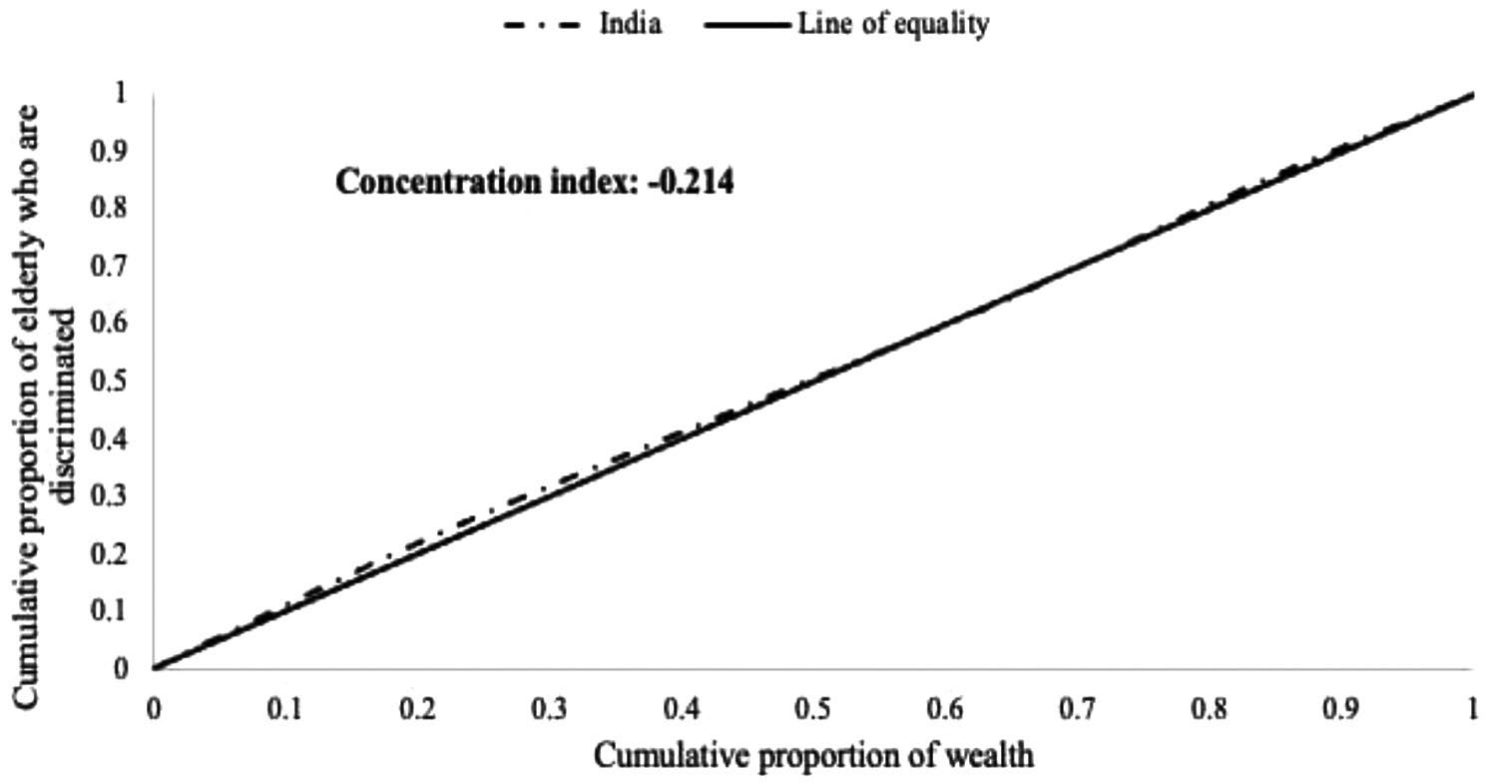
Concentration curve and index for discrimination index among 60 + older adults in India

For our first research question, we hypothesized that there would be a disproportionate concentration of perceived discrimination among older adults from low economic backgrounds. The concertation index value is -0.214, and the curve lies above the line of equality, suggesting that discrimination among older adults are concentrated among the economically vulnerable older adults. Thus, there is evidence in support of our hypothesis that older adults from low economic backgrounds perceive disproportionate levels of discrimination relative to older adults from high economic backgrounds. The concentration curve for discrimination is displayed in Fig. 1.

**Fig. 2:**
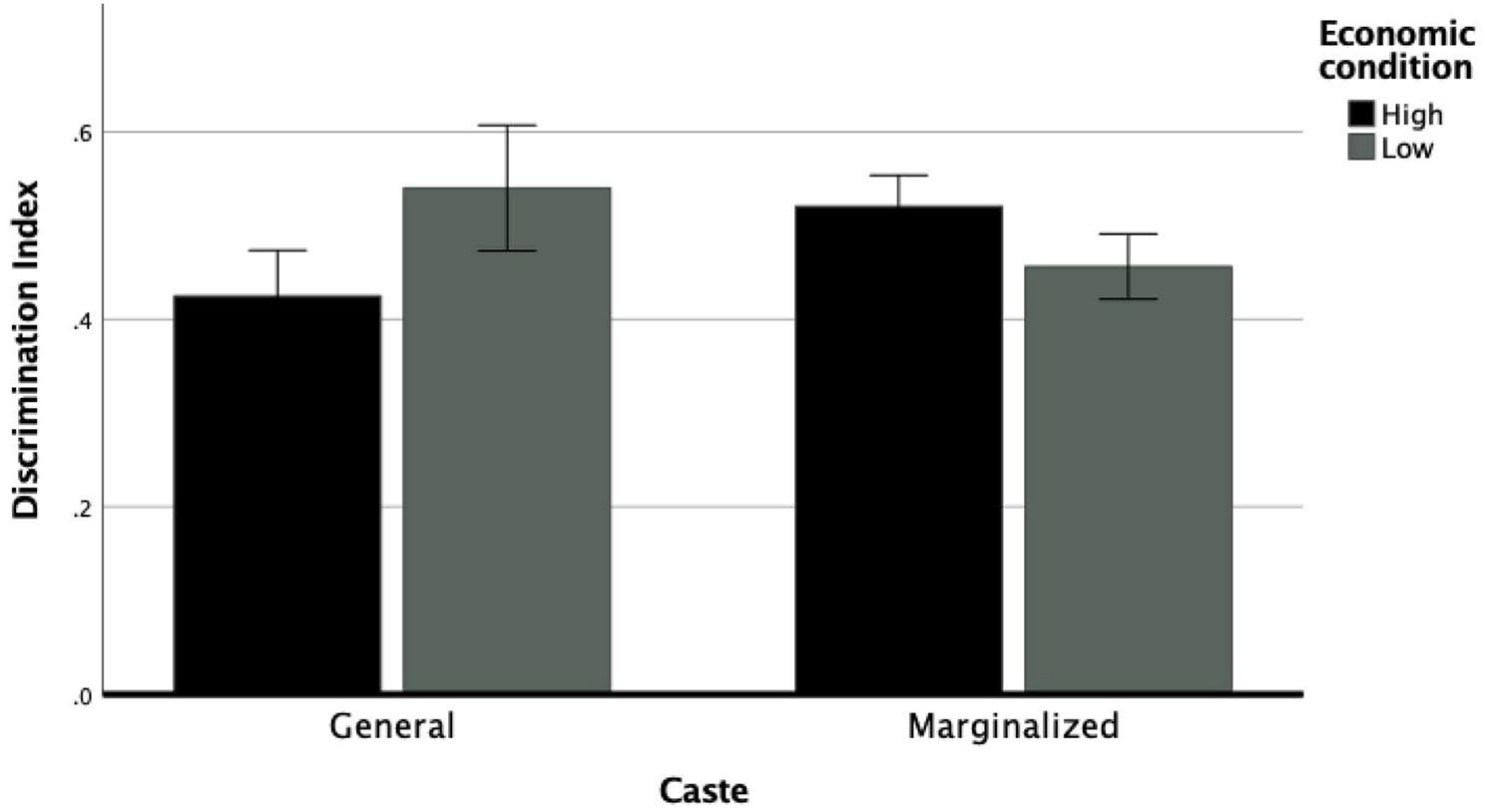
The effect of caste and economic condition on discrimination index for male-identifying individuals; *Note* Error bars represent standard error

**Fig. 3:**
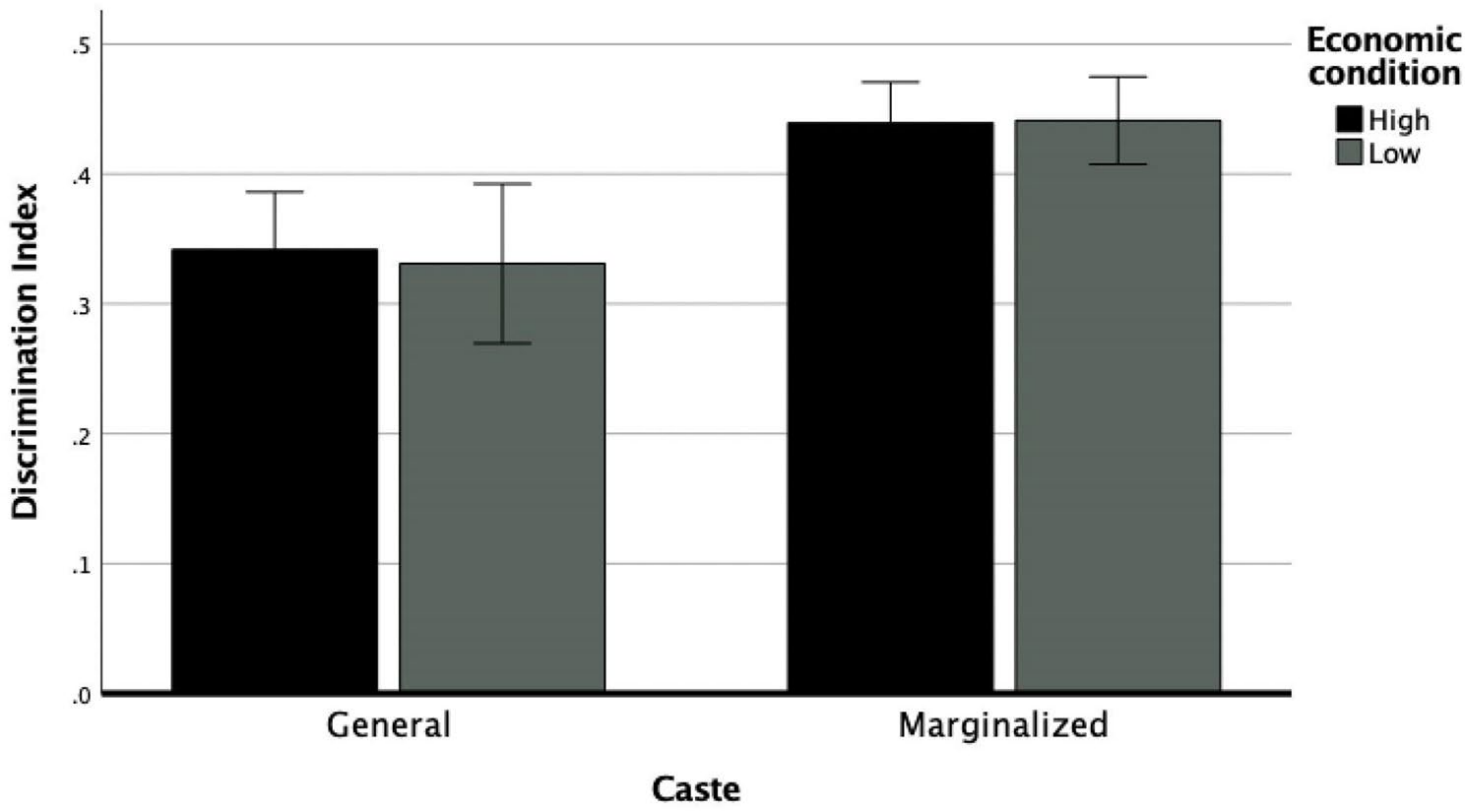
The effect of caste and economic condition on discrimination index for female-identifying individuals; *Note* Error bars represent standard error

For our second research question we hypothesized that gender, caste, and economic condition will intersect to produce the highest levels of perceived discrimination for women from lower castes and who belong to low economic backgrounds. We used a three-way ANCOVA (2 × 2 × 2 factorial design) to examine the effects of caste, gender, and economic condition on individuals’ experiences of discrimination while controlling for marital status, religious affiliation, residence, age, and education level. There was a main effect of caste, *F*(1, 30,394) = 11.15, *p* < 0.001. There was also a significant main effect of gender, *F*(1, 30,394) = 30.47, *p* < 0.001. There was no significant main effect of economic condition, *F*(1, 30,394) = 0.418, *p* = 0.52. There was a significant interaction effect between caste and economic condition, *F*(1, 30,394) = 6.80, *p* = 0.009. There was a significant interaction effect between caste and gender, *F*(1, 30,394) = 9.34, *p* = 0.002. Finally, there was a significant interaction effect between caste, gender, and economic condition, *F*(1, 30,394) = 0.8.91 *p* = 0.003 (see Figs. 2 and 3).

Post hoc analysis of the three-way interaction revealed that men from marginalized castes and of high economic condition reported greater discrimination than men of General caste and high economic condition; *t*(30,404) = 3.43, *p* < 0.001, *d* = 0.04. Women from marginalized castes and high economic condition reported greater discrimination than women of General caste and high economic conditions; *t*(30,404) = 3.63, *p* < 0.001, *d* = 0.04. On the other hand, men from General caste and low economic conditions reported higher discrimination than men from marginalized castes and low economic conditions; *t*(30,404) = 3.11, *p* = 0.026, *d* = 0.04. Women from marginalized castes and low economic conditions reported greater discrimination than women of General caste and low economic conditions; *t*(30,404) = 3.24, *p* = 0.001, *d* = 0.04.

Men from high economic conditions and from General caste reported greater discrimination than high economic condition women from General caste; *t*(30,404) = 2.56, *p* = 0.011, *d* = 0.03. Men from low economic condition and from General caste also reported greater discrimination than low economic condition women from General caste; *t*(30,404) = 4.54, *p* < 0.001, *d* = 0.05. Similarly, men from high economic condition and from marginalized castes reported greater discrimination than high economic condition women from marginalized caste; *t*(30,404) = 3.52, *p* < 0.001, *d* = 0.04. Men from marginalized caste and of low economic condition also reported higher discrimination than women of marginalized castes and of low economic condition but this difference did not reach statistical significance; *t*(30,404) = 0.625, *p* = 0.53, *d* = 0.007.

General caste men from high economic conditions reported greater discrimination than general caste men from low economic conditions; *t*(30,404) = 2.80, *p* = 0.005, *d* = 0.03. General caste women from high economic conditions reported greater discrimination than general caste women from low economic conditions but the difference did not reach statistical significance; *t*(30,404) = 3.43, *p* < 0.001, *d* = 0.04. Marginalized caste men from high economic conditions reported higher discrimination than marginalized caste men from low economic conditions; *t*(30,404) = 2.66, *p* = 0.007, *d* = 0.03. Marginalized caste women of low economic conditions reported greater discrimination than marginalized caste women of high economic conditions, but the difference was not statistically significant; *t*(30,404) = 0.09, *p* = 0.933, *d* = 0.01.

Our third hypothesis was that education plays a moderating role between caste and experiences of discrimination in that higher education leads to lesser experiences of discrimination. We conducted a moderation analysis (see Table 3) where we have analysed the relationship between caste (X) and discrimination (Y), moderated by education (M). The regression coefficients (β) and standard errors (SE) have been reported. Belonging to marginalized groups, that is, SC/ST/OBC, predicted higher discrimination (β = 0.104; *t*(30,404) = 4.522; *p* < 0.001) in comparison to General castes. The negative association of perceived discrimination with education, indicates that with increasing levels of education, there are lesser chances of discrimination (β= -0.124; *t*(30,404)= -8.267; *p* < 0.001). The moderating effect of education is indicated by the interaction effect of caste and education. Belonging to marginalized caste groups, that is, SC/ST/OBC, and with increasing levels of education, the chances of discrimination decreases (β= -0.192; *t*(30,404)= -14.769; *p* < 0.001) in comparison to General caste. The model was controlled for other covariates, which indicated that, females (β = 0.068; *t*(30,404) = 4.533; *p* < 0.001), with increasing age (β = 0.180; *t*(30,404) = 8.571; *p* < 0.001), non-Hindu minorities (β= -0.071; *t*(30,404)= -3.381; *p* < 0.01), marital status being not married/divorced/separated/widowed (β = 0.138; *t*(30,404) = 6.273; *p* < 0.05), belonging to rural residence (β = 0.028; *t*(30,404) = 2.333; *p* < 0.05), and with low economic condition backgrounds (β= -0.022; *t*(30,404)= -3.667; *p* < 0.01) have higher chances of discrimination. The moderation of education between caste and discrimination in the model thus results in a 10.2% variability in the discrimination index [*F*(13, 30,393) = 24.55; *p* < 0.001].

**Table 3:** Moderation model with reported regression coefficients (B) and standard errors (SE)

		Discrimination	
		**β**	**SE**
Constant		0.464***	0.072
Caste (X)	Others/General ®		
	SC/ST/OBC	0.104***	0.023
Education level (M)		-0.124***	0.015
Caste*Education level (X*M)	Others/General*Education level ®		
	SC/ST/OBC*Education level	-0.192***	0.013
*Covariates*			
Age		0.180***	0.021
Gender	Male ®		
	Female	0.068***	0.015
Residence	Urban ®		
	Rural	0.028*	0.012
Religion	Hindus ®		
	Non-Hindus	-0.071**	0.021
Marital status	Married ®		
	Not married	0.138***	0.022
MPCE quintile		-0.022**	0.006
*R* ^*2*^		0.102	
*F*		24.555	*p* < 0.001

*Note* SC, ST, OBC stand for Scheduled Castes, Scheduled Tribes, and Other Backward Castes, respectively; MPCE stand from Monthly per capita expenditure

***: *p* < 0.001; **: *p* < 0.01; *: *p* < 0.05

**Fig. 4:**
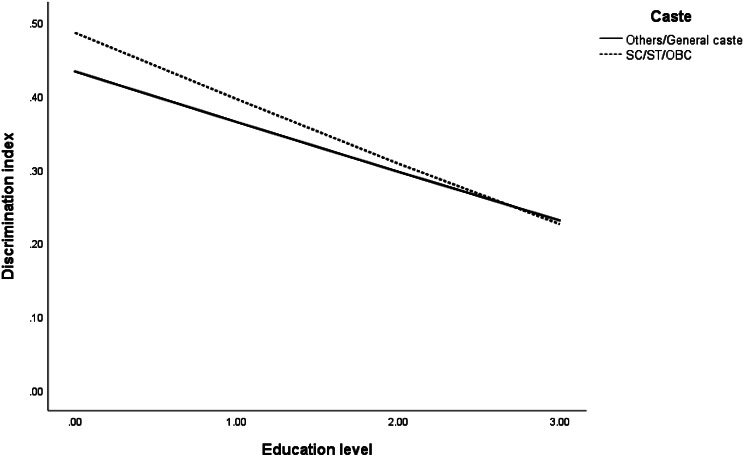
Moderating effect of education level between caste and discrimination

The moderating effects of education levels on the relationship between caste and perceived discrimination are depicted in Fig. 4. The absolute slope of the curve showing the association of one’s education and perceived discrimination is steeper downward (more negative) for marginalized castes than General castes. It implies that a person’s education level strengthens the relationship between their caste identity and perceived discrimination. With the increase in the education level, discrimination is reduced for older adults belonging to marginalized castes.

## Discussion

Results from the current study demonstrate that there is a significant concentration of perceived discrimination among older adults who classify as economically disadvantaged. Gender, caste, and economic condition interact in complex ways to contribute to unique experiences of discrimination, and this effect is robustly evidenced in women who are from low economic backgrounds and belong to a marginalized caste (thereby facing a triple disadvantage); finally, education moderates the relationship between caste and perceived discrimination in that marginalized caste folks who have received an education at the primary, secondary or diploma/college level reported lower levels of perceived discrimination. To our knowledge, this is the first study to employ an intersectional lens to analyze the drivers of discrimination in India and adds to vast the literature on harnessing education as a tool to mitigate social inequalities.

### Concentration of perceived discrimination

Discrimination is an important social determinant of health [22]. Historically, discrimination has been documented in low-income populations (e.g., Borrell et al., 2010) [71]; however, such studies have primarily documented the experiences of folks in the global north. In assessing the economic oppression faced by African Americans in the US, Cavalhieri & Wilcox (2022) found that discrimination experienced by African Americans significantly predicted stress, well-being, and depression [72]. In another study on assessing the relationship between economic condition and health [73], found that the perception of economic positioning-based discrimination in adolescents is an important mechanism behind the impact of poverty on health. Furthermore, Achdut (2023) found that subjective perceptions of economic deprivation, material deprivation, and loneliness were significant predictors of perceived discrimination in young adults in Israel [74].

The unique contribution of the current study to the pre-existing literature is the documentation of high levels of discrimination amongst older adults in India who classify as economically disadvantaged. The representation of older Indian adults is crucial to lend coherence to the narrative of economic marginalization as a lived reality in India, a developing nation with a steadily increasing wealth gap between the rich and the poor (see Ghatak et al., 2022, for a review of wealth gap inequality in India) [75]. Such narrative coherence is fundamental to advocating for targeted poverty alleviation measures through policymaking and for combating essentialist beliefs about poverty that perpetuate discrimination in a vicious cycle. For example, an essentialist belief about poverty is that people living in poverty are responsible for their condition. Endorsement of this belief would lead to viewing economically disadvantaged people as inferior and not deserving of assistance. A gradual accumulation of such endorsement would ultimately lead to a master narrative of otherization that promulgates poverty as a personal ill that does not warrant structural redressal. Attributing social disparities to groups’ intrinsic natures has been demonstrated to produce racial and ethnic prejudice [76, 77], endorsement of the legitimacy of male-female power inequality [78], opposition to women’s and transgender peoples’ rights [79], support for the societal status quo [80], and support for eugenics [81]. Alternatively, structural accounts of poverty lead to viewing poverty as a systemic issue, thereby shaping cultural schemas about poverty [82] that drive social action.

### Gender, caste, economic condition

Results from the current study evidenced some interesting patterns regarding the interplay of gender, caste, and economic condition on perceived discrimination in older Indian adults. First, the finding that women who are economically disadvantaged and belong to a marginalized caste face greater discrimination (triple jeopardy; see Greene, 1996, p. 389–427 for more on triple jeopardy) [83] than women of General caste and low economic condition. This finding lends support to the idea of General caste as a protective factor in experiences of discrimination in older Indian women. Caste is a strong driver of discrimination and violence against women in India. Schedule caste women experience higher odds of lifetime physical abuse than women of other castes [84]. Schedule caste women are also more likely to experience domestic violence [85]. In recent years, caste-based atrocities in India against economically disadvantaged marginalized caste women have sky-rocketed insofar as the BBC reported, “Dalit women are among the most oppressed in the world” [86]. It is noteworthy to factor in the role of economic condition in the context of the oppression faced by Dalit women. Since caste and economic condition share a bidirectional relationship, Dalit women are often economically disadvantaged. They face the “triple jeopardy” of gender bias, caste discrimination, and economic deprivation. This is the nature of intersectional oppression we aspired to empirically demonstrate through our study. The current results concur with accounts of oppression faced by triply oppressed marginalized caste women in India, thereby necessitating public and policy-level discourse on combating gender, caste, and economic condition-based discrimination as a whole rather than isolated units.

Contrary to our hypothesized expectations that women would overall report the highest levels of discrimination than men (of different caste and economic conditions ), the current results demonstrate that General caste men reported greater levels of discrimination than marginalized caste men, General caste women from high and low economic conditions, and marginalized caste women from high and low economic conditions. These findings call into consideration who considers themselves to be victims of discrimination. Kobrynowicz & Branscombe (1997) found that men who had low self-esteem and high personal assertiveness reported higher levels of personal discrimination [87]. In their findings, low self-esteem was also related to men’s perception of discrimination as a group. In contrast, for women, a high need for approval was negatively related to perceptions of discrimination, while depression was positively related. Considering these findings, the current results contribute to the literature that perceptions of discrimination serve different purposes for structurally privileged and disadvantaged groups. General caste men who are economically privileged might report higher levels of discrimination due to low self-esteem, which serves as a driver of preserving patriarchy. Indeed, feelings of powerlessness, discrimination, and experiences of limited self-esteem and self-confidence jointly contribute to the subordination of women in a patriarchal society [88].

### Is education the great equalizer?

The current results support the hypothesis that education moderates the relationship between caste and perceived discrimination, although it must be noted that the results demonstrated a partial mediation. This essentially means that caste status had a direct effect on perceived discrimination, and that relationship was partly ameliorated by education. Therefore, marginalized caste folks who have received an educational degree reported lower levels of perceived discrimination than folks who did not receive any education. The reason behind this can be manifold. Education serves as a tool for upward social mobility, especially for women of minority caste groups in India [89]. Upward social mobility presents opportunities that permit access to spaces that were hitherto inaccessible. Such experiences may lead to perceptions of equal treatment [59] that serve as a buffer between caste affiliation and perceived discrimination. Alternatively, access to spaces that were historically reserved for the privileged may lead to feelings of ‘being ashamed’ of one’s group of origin and identifying more with the privileged group, thereby leading to better acculturation with the new group, ultimately experiencing lesser discrimination. However, Naudet (2008) found that in the context of Dalits in India, this is unlikely to be the case, as Indian Dalits’ upward mobility is shaped by the perpetuation of a link with their group of origin in the hope of ‘paying back to society’ [90].

**Fig. 5:**
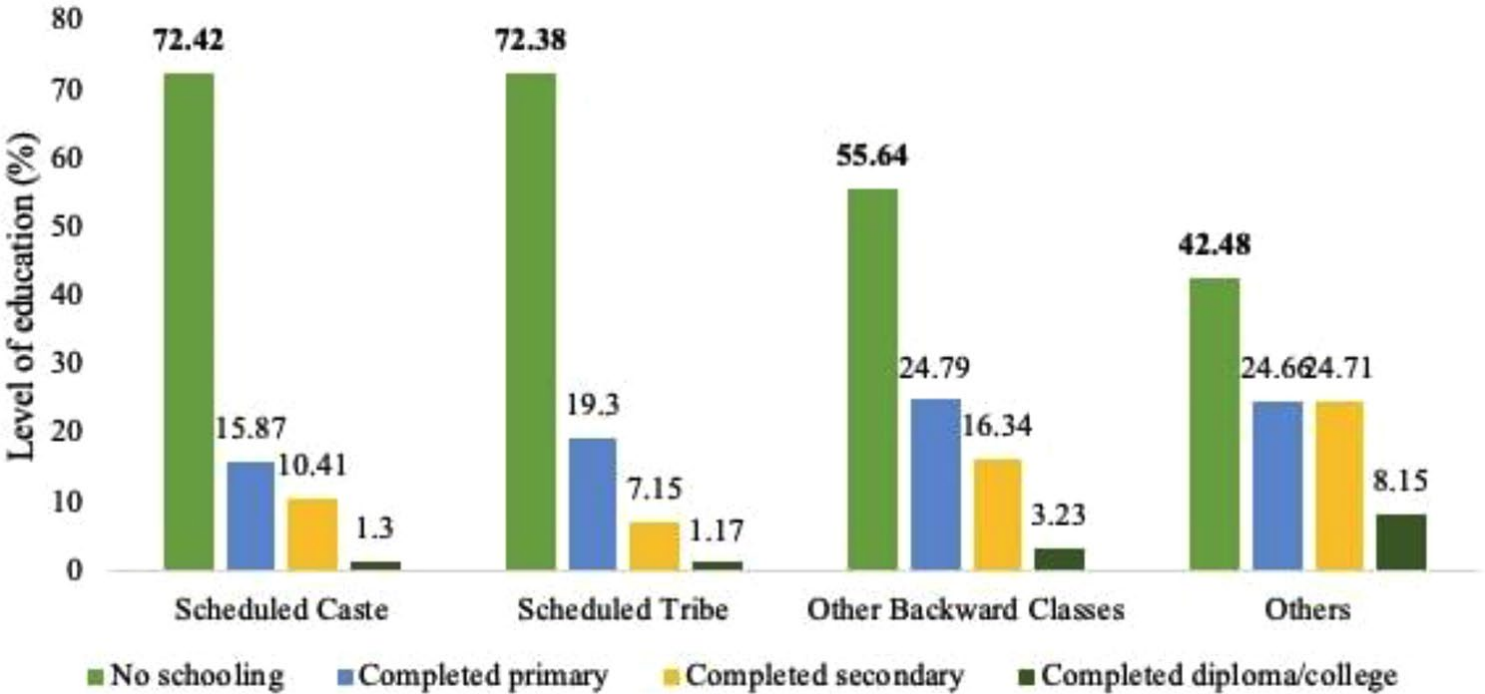
Caste-wise percentage distribution of level of education among older adults

An alternative reality in educational attainment by minoritized groups is an increased awareness of higher levels of perceived discrimination. This has been termed the “paradox of perceived discrimination” by Gelepithis & Giani (2021) [91]. However, this phenomenon was not evidenced in our model. A potential reason could be that the quality of education, and not just quantity (measured by years of schooling; see Majumdar 2009, for debates on quality versus quantity of education in the Indian context)leads to the increased awareness of discrimination [92]. Although around 28% of ST, SC, and 44% of OBC folks in our sample had some level of education (Fig. 5), it does not speak about the quality of education. Alternatively, another possibility could be related to our measure of perceived discrimination. It is plausible that our measure of perceived discrimination did not capture the experiences of discrimination faced by marginalized folks in the workspace, which necessitates the development of more measures and future research that measure experiences of discrimination with a context, situation, and culture-specific nuance.

Finally, it should be noted that we tested out a moderation model with caste status predicting perceived discrimination and this relation being moderated by levels of educational attainment. This directionality should be considered in interpreting the results. An alternative directionality could be that experiences of discrimination impact access to education for individuals from marginalized castes. This directionality implies that discrimination may serve as a barrier to educational opportunities rather than educational attainment, influencing perceptions of discrimination. This necessitates future investigation.

### Limitations

The current study is not without limitations. We analyzed three drivers of discrimination in India, namely, gender, caste, and economic condition. Some other salient conduits of discrimination in the Indian context are religious identity, ethnic identity, language, marital status, citizenship status, to name a few. Therefore, future research must strive to capture the intersection of drivers of discrimination beyond the tripartite structure of gender, caste, and economic condition. Additionally, having people above 60 in age in the sample, might reflect a bias of excluding members of groups with a lower life expectancy. However, in India 60, is usually taken as a standard old age threshold since, in most sectors, it is the retirement age [93]. In another vein, the current sample comprised older adults whose experiences of discrimination might be different from the experiences of younger generations of India who are growing up in an era of rising religious majoritarianism, hate crimes, and caste-based otherization. As of July 2023, India ranks eighth among the countries that are at the highest risk for genocide [94]. The study also has limitations owing to the cross-sectional nature of the data, based on which causality cannot be established. The self-reported responses of the older adults can also cause biases in measuring perceived discrimination. Future research could potentially investigate the interrelation between perceived and actual discrimination.

## Conclusion

In sum, the current paper advocates for employing an intersectional lens in documenting perceived discrimination. Importantly, an intersectional lens is also crucial to understanding the unique ways in which older adults in India demonstrate resilience in the face of oppression. Poverty alleviation programs must factor in caste and gender in their interventional efforts. Similarly, combating caste- and gender-based discrimination should entail a socio-historical appraisal of the role of economics in individual agency.

India lacks a strong policy and legal framework to prevent such age, caste, and gender-based discrimination, which necessitates structural addressing. Special actions are required at the family, institutional, community, and government levels to eliminate day-to-day prejudice against older adults. There should be a shift in the community’s negative attitude towards the older population, to ensure their overall psychological well-being. Furthermore, health-care practitioners should be aware that discrimination is a major source of stress in old age, particularly among vulnerable populations with disadvantaged economic conditions and health status.

Finally, education should be central to conceptualizing development in India. Moreover, the policy-level focus should be on the quality of education over and above quantity. Education is certainly not the greatest equalizer in a meritocratic society, but perceptions of agency, belongingness, and equal treatment fostered by education could combat discrimination and, in the process, contribute to a stronger democracy.

## Data Availability

The analysis is based on secondary data available in public domain for research; thus, no approval was required from any institutional review board (IRB). The data is freely available upon request from https://iipsindia.ac.in/sites/default/files/LASI_DataRequestForm_0.pdf. **Point of contact** Sampurna Kundu, Email: sampurna34@gmail.com.
